# T-bet represses collagen-induced arthritis by suppressing Th17 lineage commitment through inhibition of RORγt expression and function

**DOI:** 10.1038/s41598-021-96699-5

**Published:** 2021-08-30

**Authors:** Masaru Shimizu, Yuya Kondo, Reona Tanimura, Kotona Furuyama, Masahiro Yokosawa, Hiromitsu Asashima, Hiroto Tsuboi, Isao Matsumoto, Takayuki Sumida

**Affiliations:** 1 Department of Internal Medicine, Faculty of Medicine, University of Tsukuba, 1-1-1 Tennodai, Tsukuba City, Ibaraki 305-8575 Japan; 2 Departments of Neurology and Immunobiology, Yale School of Medicine, New Haven, CT 06520 USA

**Keywords:** Immunology, Molecular biology, Rheumatology

## Abstract

T-bet is a key transcription factor for the T helper 1 lineage and its expression level is negatively correlated to inflammation in patients with rheumatoid arthritis (RA). Our previous study using T-bet transgenic mice revealed over-expression of T-bet completely suppressed collagen-induced arthritis (CIA), a murine model of RA, indicating a potential suppressive role of T-bet in the pathogenesis of autoimmune arthritis. Here, we show T-bet-deficiency exacerbated CIA. T-bet in CD4 + T cells, but not in CD11c + dendritic cells, was critical for regulating the production of IL-17A, IL-17F, IL-22, and TNFα from CD4 + T cells. T-bet-deficient CD4 + T cells showed higher RORγt expression and increased IL-17A production in RORγt-positive cells after CII immunization. In addition, T-bet-deficient naïve CD4 + T cells showed accelerated Th17 differentiation in vitro. CIA induced in CD4-Cre T-bet^fl/fl^ (cKO) mice was more severe and T-bet-deficient CD4 + T cells in the arthritic joints of cKO mice showed higher RORγt expression and increased IL-17A production. Transcriptome analysis of T-bet-deficient CD4 + T cells revealed that expression levels of Th17-related genes were selectively increased. Our results indicate that T-bet in CD4 + T cells repressed RORγt expression and function resulting in suppression of arthritogenic Th17 cells and CIA.

## Introduction

Rheumatoid arthritis (RA) is a chronic inflammatory disease characteristic of polyarthritis and, if uncontrolled, the subsequent joint destruction leads to impaired quality of life. The pathology of affected joints shows inflamed synovial membrane with leukocyte infiltration, cartilage destruction, and bone erosion. Although the precise mechanism of RA remains elusive, CD4 + T cells, especially their subsets and the transcriptional regulation of their differentiation into specific subsets, seem to play a critical role in the pathogenesis of autoimmune arthritis.

Differentiation of naïve CD4 + T cells is dependent on a lineage-specific transcription factor. For example, T-bet is the key transcription factor for T helper 1 (Th1) cells, a subset of CD4 + T cells that produce interferon-γ (IFNγ)^1^. Retinoic acid-related orphan receptor (ROR) γt is another important transcription factor for T helper 17 (Th17) cells, a subset of CD4 + T cells characterized by the production of interleukin (IL)-17A^2^. In the peripheral blood of patients with RA, the expression levels of *tbx21* (which encodes T-bet) and *ifng* negatively correlates with C reactive protein, a serum protein that represents systemic inflammation^3^. Additionally, the expression level of *rorc*, which encodes RORγt, in CD4 + T cells from the peripheral blood of patients with RA is significantly higher and the frequency of Th17 cells is increased in those with active arthritis^4^. Thus, imbalance of T-bet and RORγt leading to commitment to Th17 lineage seems to be deeply involved in the pathogenesis of autoimmune arthritis.

Collagen-induced arthritis (CIA) is a murine model of human RA induced by immunization with collagen type II (CII). Development of CIA is dependent on certain cytokines. Loss of IFNγ signaling increases the severity of CIA^5–7^, but IL-17 receptor A-deficient mice are resistant to CIA^8^. Transcriptional regulation of lineage commitment of CD4 + T cells is also important. We previously reported that T-bet transgenic (T-bet Tg) mice were resistant to CIA with a reduction of *rorc* and *ahr* expression levels of CD4 + T cells leading to impaired CII-reactive IL-17A production from CD4 + T cells^9,10^. Although T-bet seemed to have a suppressive role in CIA through Th17 cell inhibition, T-bet Tg mice had abnormality in the development of T cells in the thymus^9^, which might have impinged on the pathogenesis of CIA. Thus, the precise role of T-bet in the pathogenesis of CIA from the perspective of lineage commitment of CD4 + T cells remains to be elucidated.

To determine the role of T-bet in murine autoimmune arthritis, CIA was induced in wild-type C57BL/6 (WT) mice and T-bet knockout (T-bet KO) mice and was found to be more severe in T-bet KO mice. T-bet-deficient CD4 + T cells showed higher RORγt expression and increased IL-17A production in RORγt + cells. Expression levels of *rorc* and its target genes including *il17a* were higher in T-bet-deficient CD4 + T cells. T-bet-deficient naïve CD4 + T cells showed accelerated Th17 differentiation. CIA induced in cKO mice was more severe and the percentage of Th17 cells was higher in the arthritic joints. Transcriptome analysis of CD4 + T cells from cKO mice revealed that expression levels of Th17 cell-related genes were selectively increased. Our findings indicate that T-bet in CD4 + T cells negatively regulates autoimmune arthritis by selective inhibition of Th17 differentiation through suppression of RORγt expression and function resulting in inhibition of arthritogenic Th17 cells.

## Results

### The development of T cells in the thymus was normal in T-bet KO mice

As T-bet deficiency might affect T cell development in the thymus, we analyzed the fractions of T precursor cells in the thymus (Fig. S1). Total number of thymocytes and the proportion of T precursor cells was comparable between the two strains. This allowed for analysis of the role of T-bet in T cells with normal development.

### CIA was exacerbated in T-bet KO mice

**Figure 1:**
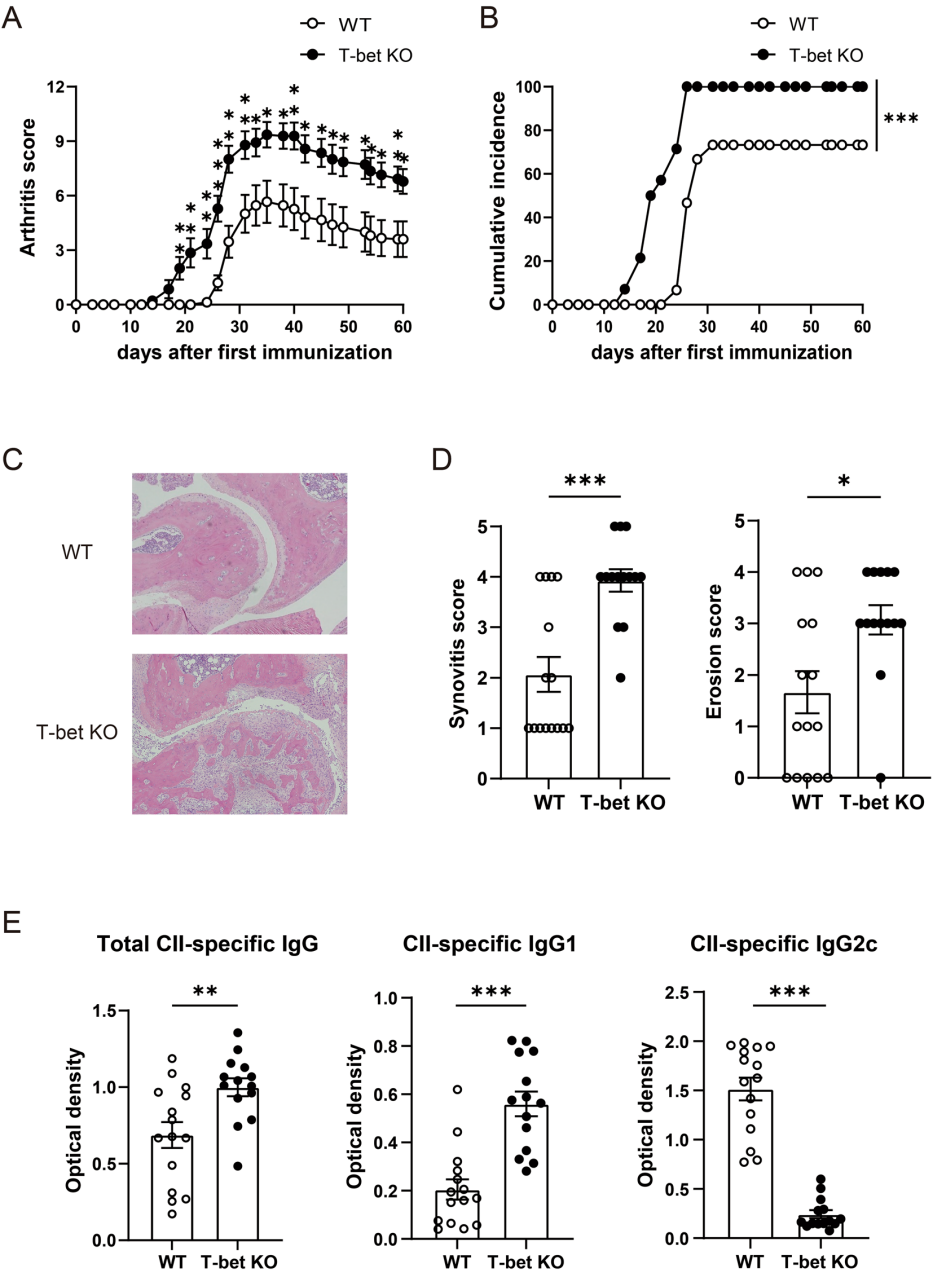
T-bet has a protective role in CIA. WT mice (n = 15) and T-bet KO mice (n = 14) were immunized intradermally with chicken CII emulsified with CFA on days 0 and 21. (**A**) Severity of CIA. (**B**) Cumulative incidence of arthritis. (**C**,**D**) At day 60 post first CII immunization, pathological severity was evaluated for decalcified hematoxylin and eosin-stained sections. Inflammation and bone erosion scores were assessed in both groups. (**E**) At day 60 post first CII immunization, serum samples were collected from WT mice (n = 15) and T-bet KO mice (n = 14) for measurement of CII-specific total IgG, IgG1, and IgG2c levels. Data are presented as mean ± SEM. Statistical tests: Welch’s t test (**A**,**C**,**D**), and Log-rank test (**B**). (*P < 0.05, **P < 0.01, ***P < 0.001).

To evaluate the role of T-bet on the development and pathogenesis of arthritis, we compared the severity and incidence of CIA in T-bet KO mice and WT mice. The arthritis induced in T-bet KO mice was markedly more severe compared with WT mice (Fig. 1A). Similarly, the incidence was also significantly higher in T-bet KO mice than WT mice (*P* < 0.001) (Fig. 1B). Histological analysis of arthritic joints showed marked infiltration of inflammatory cells (*P* < 0.001) and widespread bone erosion (*P* = 0.01) throughout joint tissues of T-bet KO mice, but those of WT mice was milder (Fig. 1C,D). The levels of total CII-specific IgG (*P* = 0.006) and IgG1 (*P* < 0.001) were significantly higher, but those of IgG2c (*P* < 0.001) were lower in T-bet KO mice than WT mice, as determined using ELISA (Fig. 1E). These results indicated that T-bet had a suppressive role in CIA.

### T-bet-deficient CD4 + T cells showed increased RORγt expression and Th17 cell-related cytokine production independent of T-bet expression in dendritic cells

**Figure 2:**
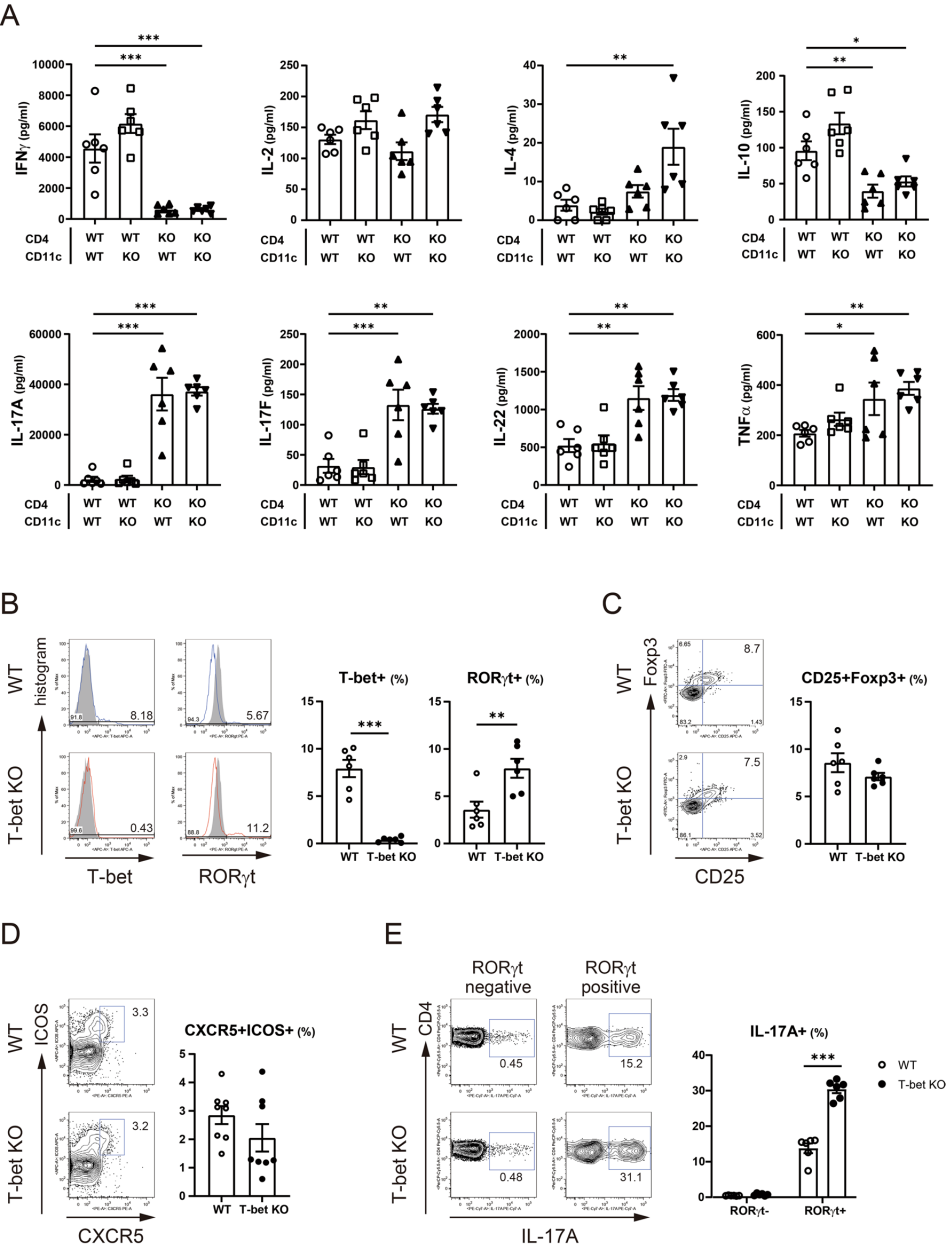
T-bet in CD4 + T cells was critical for the regulation of cytokine production from CD4 + T cells and T-bet suppressed expression and function of RORγt. At day 10 post first CII immunization, CD4 + T cells and CD11c + dendritic cells were purified from the draining LNs of WT mice (n = 6–8) and T-bet KO mice (n = 6–8) and were cultured with 100 µg/mL of denatured CII for 96 h. (**A**) Levels of the cytokines in supernatants were measured using multiplex cytokine assay. The origin of CD4 + T cells or CD11c + dendritic cells is indicated below the bar graphs. (**B**–**D**) Expression of T-bet, RORγt, Foxp3, CD25, CXCR5, and ICOS in CD4 + T cells was analyzed using FACS. For intracellular staining of cytokines, isolated CD4 + T cells from the draining LNs were stimulated with 50 ng/ml of phorbol myristate acetate and 1 μg/ml of ionomycin. (**E**) IL-17A expression in CD4 + T cells was analyzed using FACS. Data are presented as mean ± SEM. The FACS plots were generated using FlowJo (version 8.8.7, https://www.flowjo.com/), and the bar graphs were generated using GraphPad Prism 9 (version 9.1.2, https://www.graphpad.com/scientific-software/prism/). Statistical tests: one-way ANOVA with Dunnett’s post hoc test (**A**), Welch’s t test (**B**–**D**), and Welch’s t test with Bonferroni correction (**E**). (*P < 0.05, **P < 0.01, ***P < 0.001).

Since previous study reported that CIA was a Th17 cell-driven disease, we speculated T-bet deficiency might deteriorate the severity of CIA through enhanced Th17 cell response against CII. Because T-bet was expressed in CD11c + DCs after CII immunization (Fig. S2), we also evaluated the effect of T-bet in CD11c + DCs on cytokine production from CD4 + T cells using multiplex cytokine assay (Fig. 2A and Fig. S3). Production of the cytokines from WT CD4 + T cells was not affected by T-bet in CD11c + DCs. Production of IFNγ and IL-10 from T-bet-deficient CD4 + T cells was lower, but production of Th17 cell-related cytokines, namely IL-17A, IL-17F, IL-22, and TNFα from T-bet-deficient CD4 + T cells was higher independent of T-bet in CD11c + DCs, compared to WT CD4 + T cells cocultured with WT CD11c + DCs. IL-4 production from T-bet-deficient CD4 + T cells was higher than WT CD4 + T cells cocultured with WT CD11c + DCs only when cocultured with T-bet-deficient CD11c + DCs (*P* = 0.001). Intriguingly, production of IL-10 from T-bet-deficient CD4 + T cells was lower than WT CD4 + T cells. Regarding lineage commitment from the perspective of the transcription factors or cell surface markers, fluorescence activated cell sorter (FACS) analysis of CD4 + T cells from draining LNs revealed an increased percentage of RORγt + cells (*P* = 0.008) and a comparable percentage of CD25 + Foxp3 + or CXCR5 + ICOS + cells in T-bet KO mice after CII immunization (Fig. 2B–D). In addition, the percentage of IL-17A + cells was low and comparable in RORγt-negative fraction, but the percentage of IL-17A + cells was significantly higher in T-bet KO mice in RORγt + fraction (*P* < 0.001) (Fig. 2E).

Overall, these results imply that T-bet in CD4 + T cells is critical in the regulation of cytokine production, particularly Th17 cell-related cytokine production and represses not only expression, but also function of RORγt in CD4 + T cells.

### T-bet-deficient CD4 + T cells showed higher expression levels of *rorc* and its target genes including *il17a*, *il17f*, and *il22* after CII immunization

**Figure 3:**
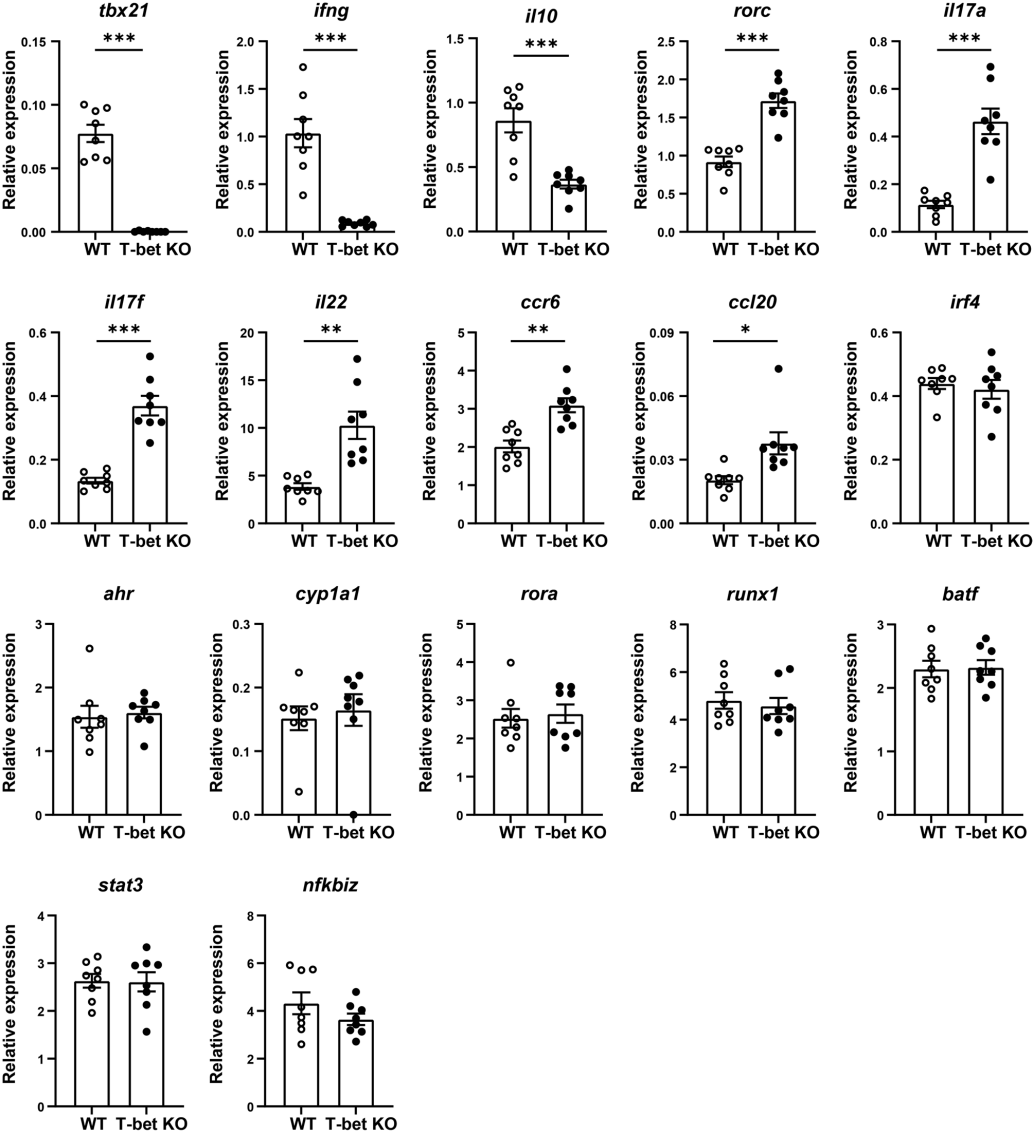
T-bet suppressed expression of *rorc* and its target genes including *il17a*, *il17f*, and *il22* in CD4 + T cells. At day 10 post first immunization with CII, CD4 + T cells were isolated from the draining LNs of WT mice (n = 8) and T-bet KO mice (n = 8). The expression levels of the genes were analyzed using qRT-PCR. Data are presented as mean ± SEM. Statistical tests: Welch’s t test. (**P < 0.01, ***P < 0.001).

We investigated effects of T-bet deficiency on gene expression levels related to Th1 or Th17 cells in CD4 + T cells after CII immunization (Fig. 3). The expression levels of *tbx21* (*P* < 0.001), *ifng* (*P* < 0.001), and *il10* (*P* < 0.001) was lower in T-bet-deficient CD4 + T cells. Consistent with the above result, the expression levels of *rorc* (*P* < 0.001) and its target genes, including *il17a* (*P* < 0.001)*, il17f.* (*P* < 0.001), and *il22* (*P* = 0.003) were higher in T-bet-deficient CD4 + T cells. The expression levels of *ccr6* (*P* = 0.001) and *ccl20* (*P* = 0.01) were also elevated. However, expression levels of the genes which are known to be involved in Th17 differentiation such as *irf4*, *ahr*, and *rora* were also comparable.

### T-bet-deficient CD4 + T cells showed a higher percentage of IL-17A + cells with a comparable percentage of RORγt + cells to WT CD4 + T cells when cultured under Th17 polarizing condition

**Figure 4:**
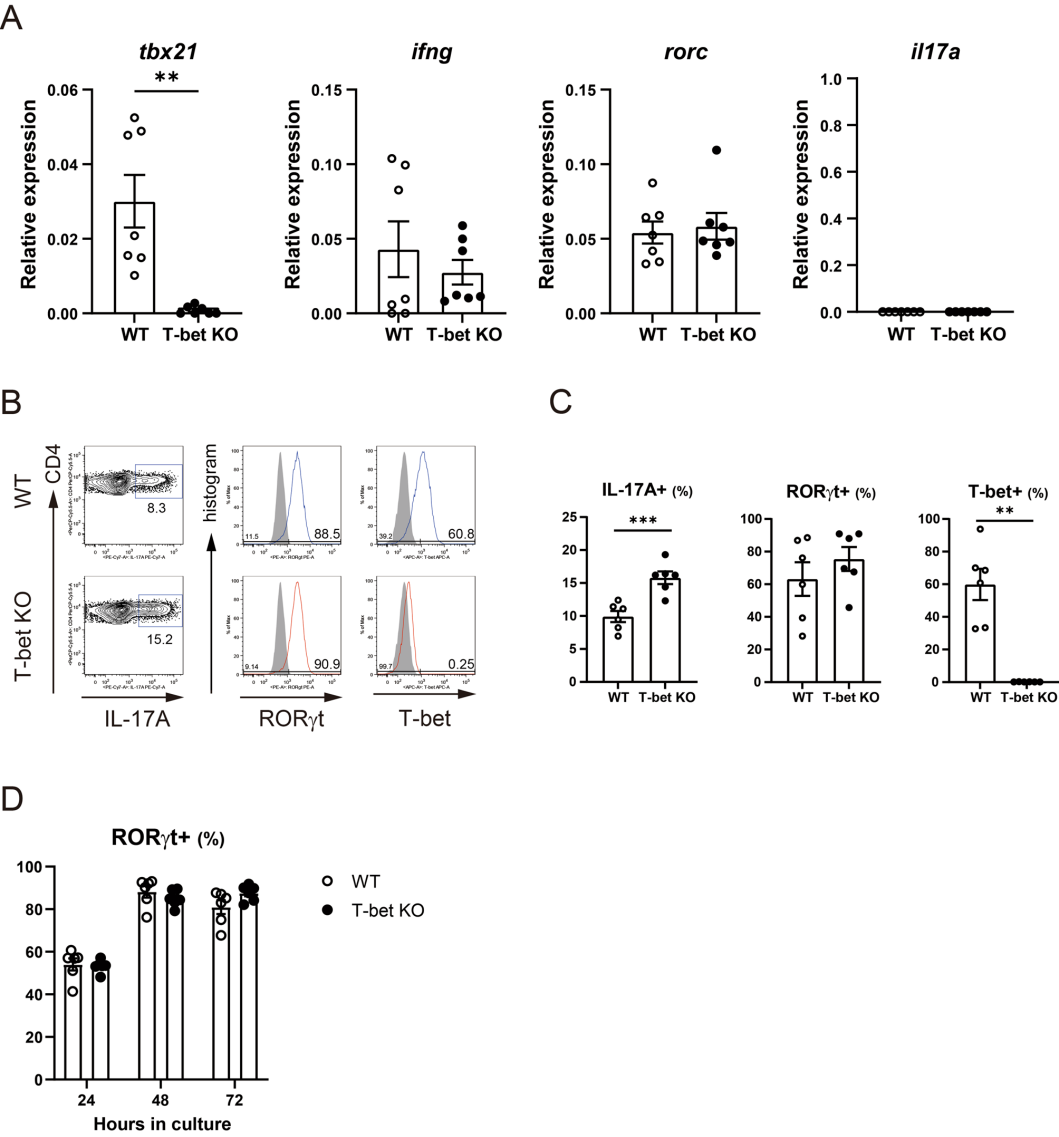
T-bet suppressed Th17 differentiation in vitro. Naïve CD4 + T cells from WT mice (n = 6–7) and T-bet KO mice (n = 6–7) were isolated. (**A**) Gene expression levels were analyzed using qRT-PCR. (**B**–**D**) Naïve CD4 + T cells were cultured under Th17 polarizing conditions and the percentage of IL-17A + cells, RORγt + cells, and T-bet + cells was analyzed using FACS. Data are presented as mean ± SEM. (**A**,**C**,**D**) was generated using GraphPad Prism 9 (version 9.1.2, https://www.graphpad.com/scientific-software/prism/), and (**B**) were generated using FlowJo (version 8.8.7, https://www.flowjo.com/). Statistical tests: Welch’s t test (**A**,**C**). (**P < 0.01, ***P < 0.001).

We evaluated whether T-bet deficiency promotes Th17 differentiation in vitro. The expression levels of *rorc* or *il17a* in naïve CD4 + T cells were comparable between the two strains excluding a possibility that T-bet deficiency had some effects on Th17 cell-related gene expression levels before Th17 polarization (Fig. 4A). When cultured under Th17 polarizing condition, T-bet-deficient CD4 + T cells showed a significantly higher percentage of IL-17A + cells (*P* < 0.001). We did not observe a substantial difference in the percentage of RORγt + cells over time (Fig. 4B–D). These results show that T-bet inhibits Th17 differentiation without repressing the expression of RORγt in vitro, which is consistent with the results of the previous ex vivo experiment which revealed that T-bet inhibited IL-17A production from RORγt + CD4 + T cells after CII immunization.

### CIA was exacerbated in CD4-Cre T-bet^fl/fl^ mice with a higher percentage of Th17 cells infiltrating into the arthritic joints

**Figure 5:**
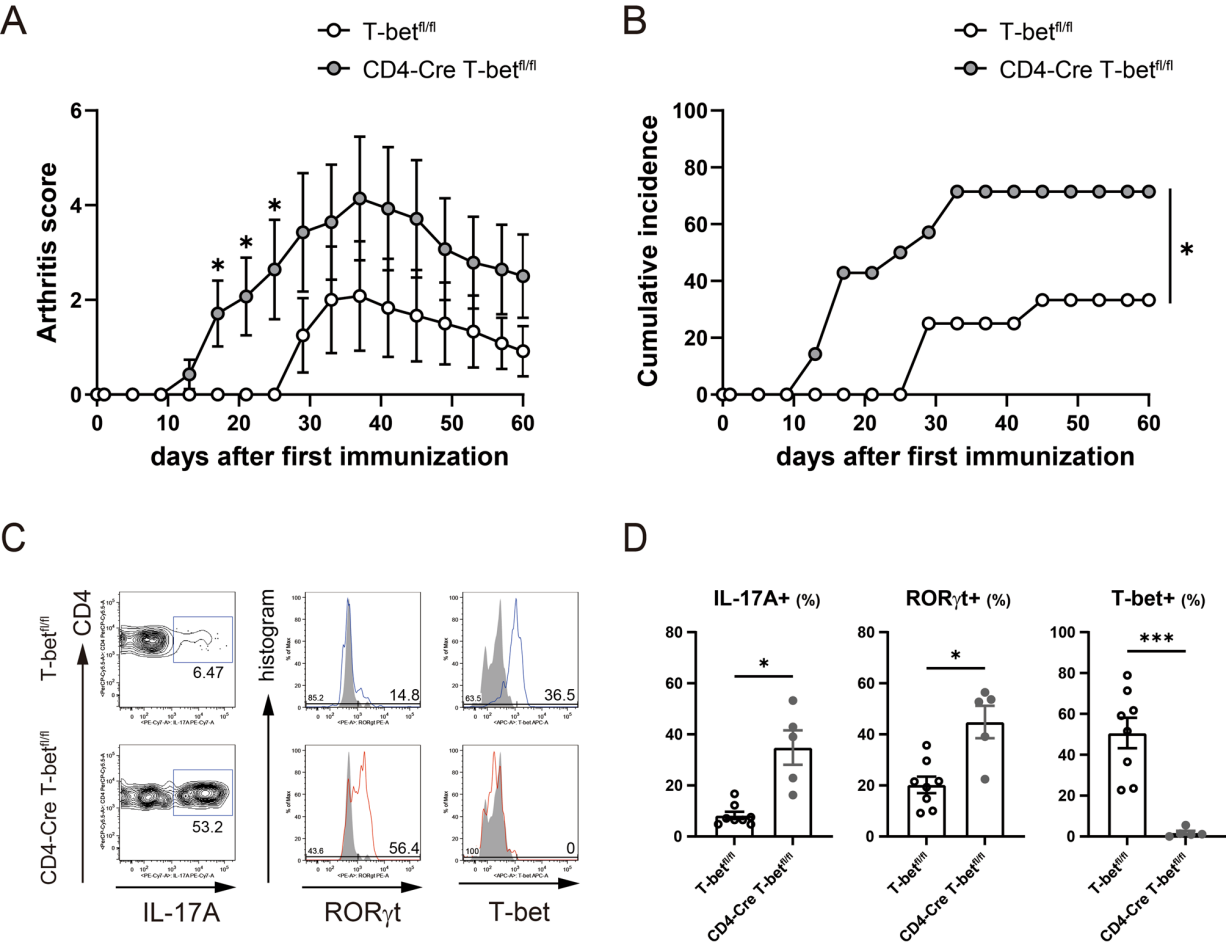
CIA in CD4-Cre T-bet^fl/fl^ mice was exacerbated and CD4 + T cells in the arthritic joints of cKO mice showed higher RORγt expression and increased IL-17A production. T-bet^fl/fl^ mice (n = 12) and CD4-Cre T-bet^fl/fl^ (cKO) mice (n = 14) were immunized intradermally with chicken CII emulsified with CFA on days 0 and 21. (**A**) Severity of CIA. (**B**) Cumulative incidence of arthritis. Ankle joints with active arthritis at day 35 post first immunization were removed from T-bet^fl/fl^ mice (n = 8) and cKO mice (n = 5) (**C**,**D**) The percentage of IL-17A, RORγt, or T-bet + cells. Data are presented as mean ± SEM. Statistical tests: Welch’s t test (**A**,**C**), and Log-rank test (**B**). (*P < 0.05, ***P < 0.001).

To evaluate the role of T-bet in CD4 + T cells on the pathogenesis of arthritis, we generated T-bet conditional KO (cKO) mice by crossing CD4-Cre mice with T-bet^fl/fl^ mice (which selectively lose the T-bet gene in T cells) (Fig. S4), and compared the severity of CIA in T-bet^fl/fl^ control mice and cKO mice. The severity of arthritis induced in cKO mice was significantly more severe and its incidence was also significantly higher than that of T-bet^fl/fl^ mice (*P* = 0.02) (Fig. 5A,B). FACS analysis of infiltrating cells into the arthritic joints revealed that RORγt expression (*P* = 0.01) and IL-17A production (*P* = 0.02) was significantly increased in T-bet-deficient CD4 + T cells of cKO mice in comparison with T-bet^fl/fl^ mice in which T-bet was expressed 51 ± 7.5% of CD4 + T cells (Fig. 5C,D). These results indicate that T-bet in CD4 + T cells is critical for the regulation of arthritogenic Th17 cells.

### RNA sequencing analysis revealed T-bet in CD4 + T cells selectively inhibited expression of Th17 cell-related genes, but not other subsets of CD4 + T cells

**Figure 6:**
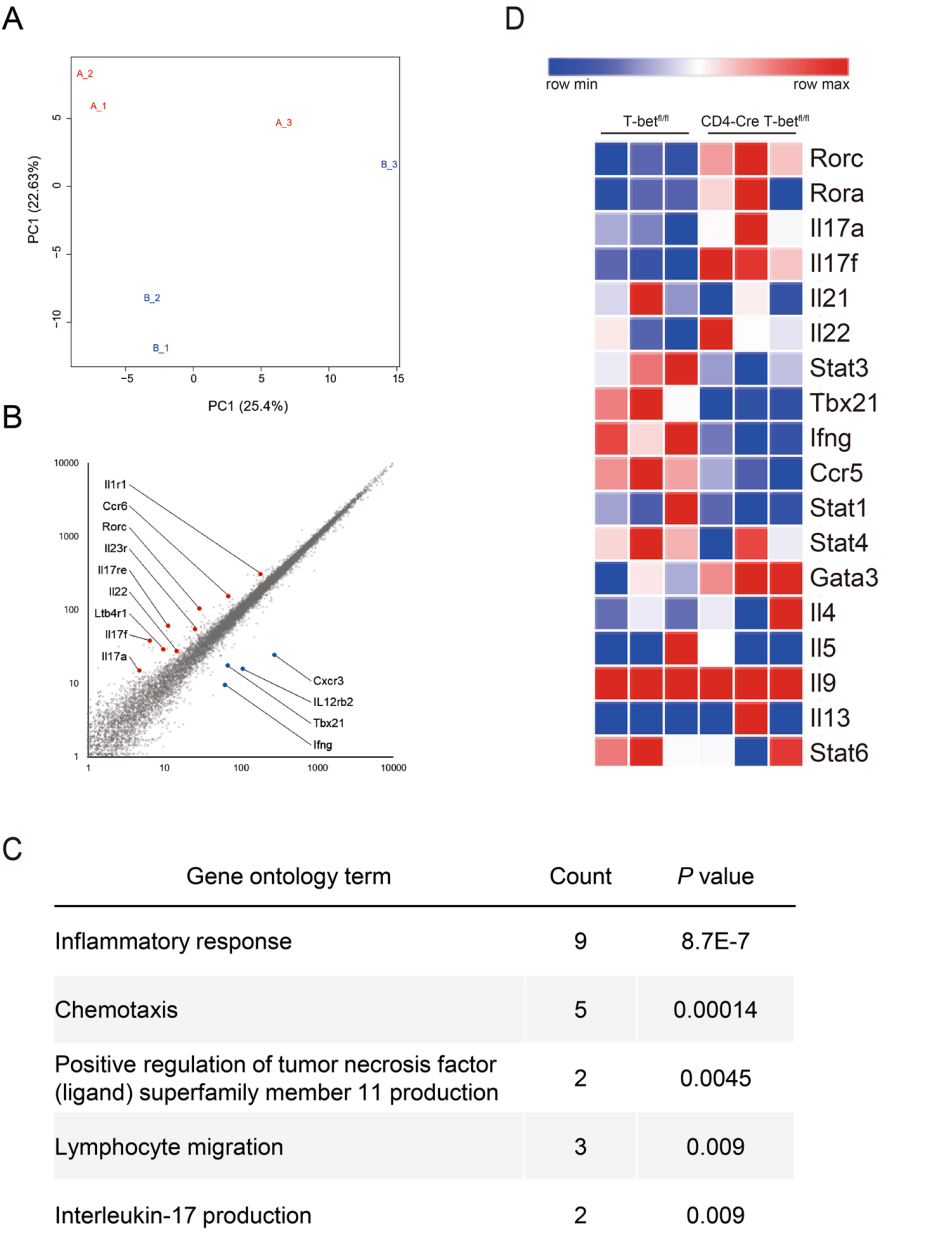
RNA sequencing revealed T-bet selectively inhibited expression of Th17 cell-related genes, but not other subsets of CD4 + T cells. At day 10 post first immunization with CII, CD4 + T cells were isolated from the draining LNs of T-bet^fl/fl^ mice (n = 3) and CD4-Cre T-bet^fl/fl^ (cKO) mice (n = 3) and the transcriptome was analyzed. (**A**) Principal component analysis of RNA-seq data. Each point represents one sample with blue points indicating T-bet^fl/fl^ mice and red points cKO mice. (**B**) Scatter plot. (**C**) GO analysis of genes differentially expressed in CD4 + T cells from T-bet^fl/fl^ and cKO mice. (**D**) Heatmap of Th cell signature genes.

To clarify the change in transcriptome of CD4 + T cells caused by T-bet deficiency, we performed RNA sequencing and profiled the transcriptome in CD4 + T cells of T-bet^fl/fl^ and cKO mice. We observed different gene expression between the two strains (Fig. 6A). T-bet deficiency in CD4 + T cells altered the expression of 52 genes with fold change more than 2.0 and false discovery rate (FDR) adjusted *P*-value of less than 0.05. Up-regulated 29 genes included several Th17 cell-related genes, while 23 down-regulated genes included several Th1 cell-related genes in cKO mice (Fig. 6B). Gene Ontology (GO) analysis of the differentially expressed genes showed a significant enrichment for inflammatory response, lymphocyte migration, and IL-17 production (Fig. 6C). Since T-bet deficiency might alter expression of other Th cell subsets, we generated a heatmap that included many of the Th cell signature genes^11^. The heatmap indicated decreased expression of Th1 cell-related genes and increased expression of Th17 cell-related genes. Intriguingly, the expression levels of Th2 cell-related genes were comparable between the two strains (Fig. 6D and Fig. S5). Taken together, these results show that T-bet in CD4 + T cells selectively inhibits Th17 cell-related genes, but not other subsets of CD4 + T cells. We searched our RNA-seq dataset of CD4 + T cells for the genes responsible for the enhanced commitment to the Th17 lineage by T-bet deficiency. Although transcriptional regulation of Th17 cells and the related transcription factors were extensively studied and reviewed in the literature^12–14^, we found no difference in the expression levels of the candidate genes (Fig. S6).

## Discussion

T-bet is the master transcription factor of Th1 lineage and promotes the expression of *ifng*, which is the signature cytokine of Th1 cells^1^. Previous studies suggested that over-expression of T-bet or IFNγ suppressed murine autoimmune arthritis^5–7,9^ and that T-bet or IFNγ negatively correlated with inflammation in RA^3^. In this study, we discovered that T-bet in CD4 + T cells repressed expression and function of RORγt and inhibited development of arthritogenic Th17 cells and pathogenesis of CIA.

We propose three possible mechanisms for T-bet-mediated regulation of CIA: inhibition of CII-reactive Th17 cells, suppression of autoantibody formation, or up-regulation of regulatory T (Treg) cells. Previous studies suggested that CD4+ T cells had different properties in the pathogenesis of CIA depending on their lineage: Th1 cells^5–7^, or Treg cells^15,16^ had a suppressive role, while Th17 cells^17^ or T follicular helper (Tfh) cells^18,19^ had a pathogenic role in CIA. Our results showed that T-bet-deficient CD4+ T cells produce a larger amount of Th17 cell-related cytokines with a smaller amount of IFNγ and IL-10, whose transcription is promoted by T-bet^20^. This cytokine production profile of T-bet-deficient CD4+ T cells was compatible with the previous studies which showed T-bet was critical for commitment to the Th1 lineage and their production of IFNγ, which regulated susceptibility to CIA through suppression of IL-17A^7^, and also indicated T-bet inhibited pathogenic potential of Th17 cells by promoting IL-10 production^21,22^. Consequently, our results strongly suggest that T-bet regulates autoimmune arthritis via suppression of commitment to the Th17 lineage. Next, titers of serum CII-specific IgG, which correlated with the severity of arthritis^23^, were higher in T-bet KO mice. Although CXCR5+ICOS+ Tfh cells positively regulated CII-specific antibody formation^19^ and their development was suppressed by T-bet^24^, the percentage of Tfh cells was equivalent between WT and T-bet KO mice. Meanwhile, in addition to smaller IFNγ and larger Th17 cell-related cytokine production, T-bet-deficient CD4+ T cells produced a mildly larger amount of Th2 cell-related cytokines, which was compatible with the previous study which showed that T-bet inhibited commitment to the Th2 lineage by directly silencing *il4*^25^. This was consistent with higher titers of serum CII-specific IgG1, which is increased by Th2 cells^26^ and Th17 cells^27^, and lower titers of serum CII-specific IgG2c, whose production is promoted by Th1 cells^28^, in T-bet KO mice. Finally, there was an equivalent percentage of CD25+Foxp3+ Treg cells, which were implicated in the control of CIA. Taken together, our results strongly suggest that T-bet regulates autoimmune arthritis via modulation of lineage commitment of CD4+ T cells, especially suppression of commitment to the Th17 lineage.

How does T-bet inhibit commitment to the Th17 lineage? Our results show that the expression levels of *rorc* and *il17a* in T-bet-deficient naïve CD4 + T cells is equivalent to those of WT naïve CD4 + T cells and T-bet-deficient naïve CD4 + T cells show promoted Th17 differentiation in vitro, which indicates that T-bet inhibits commitment to Th17 lineage from the beginning of Th17 differentiation. Moreover, the heatmap and GO analyses, using RNA-seq dataset of CD4 + T cells, indicate that T-bet deficiency in CD4 + T cells results exclusively in commitment to the Th17 lineage after CII immunization. Regarding the mechanism by which T-bet inhibits transcription of Th17-related genes, we formulated the following three hypotheses. The first hypothesis was that T-bet directly inhibits *il17a* expression; however, our observation that T-bet did not affect IL-17A production in RORγt-negative CD4 + T cells suggests that direct inhibition of *il17a* is unlikely. The second hypothesis was that T-bet inhibits *rorc* expression. T-bet-deficient CD4 + T cells exhibited up-regulation of not only *rorc*, but also *il17a*, *il17f.*, and *il22*, whose expression levels are directly regulated by RORγt^12,29,30^. In addition, IL-17A production from T-bet-deficient CD4 + T cells was increased in RORγt + T-bet-deficient CD4 + T cells, suggesting that T-bet also inhibited the function of RORγt in CD4 + T cells. The third hypothesis was that T-bet modulated the expression levels of known regulators of *rorc* or *il17a*; however, our RNA-seq dataset of CD4 + T cells revealed that T-bet had no effect on the expression levels of the genes responsible for regulating *rorc* or *il17a*. Overall, our findings support the notion that T-bet inhibits commitment to the Th17 lineage through the inhibition of expression and function of RORγt. The precise molecular mechanism by which T-bet inhibits the expression and function of RORγt was not identified in our research; however, our results raised the possibility that T-bet might modulate the function, but not the expression levels of the regulators of *rorc* or *il17a*. One of the mechanisms of functional modulation of the regulator of *rorc* or *il17a* is that T-bet forms a complex with RUNX1 and impairs the functions of RUNX1, which directly promotes *rorc* expression and works as a coactivator of RORγt to upregulates *il17a* expression^31^. This might explain our results to some extent.

What is the role of T-bet in CD4 + T cells in the arthritic joints? Our crisscross coculture experiment using CD4 + T cells and CD11c + dendritic cells clearly showed that T-bet in CD4 + T cells was critical in CII-reactive IL-17A production. To address the question above, we induced CIA in CD4-Cre T-bet^fl/fl^ (cKO) mice, which had T cell-specific T-bet ablation and confirmed aggravation of CIA in cKO mice. T-bet was expressed in about half of the CD4 + T cells in the arthritic joints of T-bet^fl/fl^ mice. T-bet-deficient CD4 + T cells in the arthritic joints of cKO mice showed higher RORγt expression and IL-17A production. IL-17A induces the production of chemokines, such as CXCL2^32^ and CCL20^33^ from synovial fibroblasts and enhances the accumulation of inflammatory cells including neutrophils^34^ and Th17 cells^35^, respectively. Moreover, Th17 cells express receptor activator of NF-κB ligand (RANKL) on their surface and induce RANKL from osteoblasts and synovial fibroblasts via IL-17A production in the arthritic joints^36,37^. Therefore, our results suggest that T-bet in CD4 + T cells represses joint inflammation and bone destruction as a consequence of inhibiting the development of arthritogenic Th17 cells.

However, T-bet in CD4 + T cells alone cannot entirely account for aggravation of CIA in T-bet KO mice. CIA induced in cKO mice seems to be somewhat weaker than T-bet KO mice, suggesting that T-bet in other immune cells may also have a suppressive role for autoimmune arthritis. In our study, T-bet in CD11c + DCs was dispensable for CII-reactive cytokine production from CD4 + T cells, but it was involved in cytokine or chemokine production from DCs such as IL-1α, macrophage inflammatory protein-1α (MIP-1α), or thymus- and activation-related chemokine (TARC)^38^ and might have impinged on the severity of CIA. Moreover, since T-bet positivity in CD4 + T cells seemed to be low in comparison to CD8 + T cells and comparable to CD19 + B cells in Figure S2, it was possible that T-bet in CD8 + T cells and B cells has a role in the pathogenesis of CIA. T-bet is critical for activated CD8 + T cells to differentiate into short-lived effector cells or memory precursor cells^39^. However, the role of T-bet in CD8 + T cells in CIA might be limited as CD8 + T cells are dispensable to induce CIA unlike CD4 + T cells^40,41^ though further studies are necessary. In B cells, T-bet is induced in response to signaling by Toll-like receptor 9^42^, whose agonist is included in complete freund’s adjuvant^43^, which was used in our experiment. Although exact role of T-bet in B cells remains unclear in our analysis, one study reported that T-bet in marginal zone B cells is related to IL-10 production and remission of CIA^44^. Further studies are necessary to elucidate the whole contribution of T-bet in autoimmune arthritis.

In conclusion, our observations suggest that T-bet in CD4 + T cells has a suppressive role for autoimmune arthritis through inhibition of the development of arthritogenic Th17 cells via suppression of RORγt expression and function. Thus, the T-bet-RORγt-Th17 axis would be a novel therapeutic target for autoimmune arthritis.

## Materials and methods

### Ethical approval

All experiments described in this report were performed according to the Guide for the Care and Use of Laboratory Animals at the University of Tsukuba and were approved by the Animal Ethics Review Committee of the University of Tsukuba. All experiments described in this report were also performed in accordance with ARRIVE guidelines.

### Mice

Age-matched male C57BL/6 WT mice, T-bet knockout C57BL/6 (T-bet KO) mice^45^, T-bet^fl/fl^ C57BL/6 (T-bet^fl/fl^) mice and C57BL/6 CD4-Cre T-bet^fl/fl^ (conditional knockout; cKO) mice (age: 8–11 weeks) were used in our experiments. T-bet KO mice and T-bet^fl/fl^ mice were purchased from Jackson Laboratory Co. (Bar Harbor, ME, USA). CD4-Cre mice were provided by Professor S. Takahashi (University of Tsukuba, Ibaraki, Japan). cKO mice were generated by crossing CD4-Cre mice with T-bet^fl/fl^ mice. All mice were maintained under specific pathogen-free conditions.

### Induction of collagen-induced arthritis

Induction of CIA was performed as previously described^9^. 2 mg native chicken CII (Sigma-Aldrich, MO, USA) was dissolved in 500 μl 0.01 M acetic acid and emulsified in complete freund’s adjuvant (CFA). CFA was prepared by mixing 2.5 mg heat-killed *Mycobacterium tuberculosis* (H37Ra) (Difco Laboratories, MI, USA) and 500 μl incomplete freund's adjuvant (Sigma-Aldrich, MO, USA). Mice were anesthetized with isoflurane and were intradermally injected at the base of the tail with 200 μg CII in CFA on days 0 and 21. Arthritis was evaluated visually, and findings in each paw were scored on a scale of 0–3 as follows; 0 = normal, 1 = slight swelling and/or erythema, 2 = moderate swelling, 3 = pronounced swelling and/or ankylosis. Scores for each limb were totaled (maximum score = 12). For histological assessment, mice were sacrificed 60 days post first CII immunization, and both ankle joints were removed. After fixation and decalcification, the ankle joints were cut into sections and stained with hematoxylin and eosin. Histological findings of each ankle joint were quantified by a blinded observer based on the degree of synovitis and bone erosion, as described previously^46^.

### Measurement of collagen-specific IgG titers

100 μl of 10 μg/ml CII in 0.1 M Na_2_CO_3_ was added to the wells of a 96-well plate and incubated at 4 °C overnight. The wells were washed 3 times with 300 μl of PBS containing 0.05% polysorbate 20 and incubated with 300 μl of PBS containing 1% bovine serum albumin (BSA) for 1 h. After washing the wells, 100 μl of sera diluted 5000 times with PBS containing 1% BSA was added to each well and incubated for 1 h at room temperature. After washing the wells, 100 μl of HRP-conjugated anti-mouse IgG, IgG1(Bethyl laboratories, TX, USA), or IgG2c (Abcam, Cambridge, UK) antibody diluted 5000 times with 1% BSA in PBS was added and incubated for 1 h at room temperature. After washing the wells, 100 μl of 3,3’,5,5’-tetramethylbenzidine was added to each well. After incubation for 5 to 10 min, 50 μl of 1 M H_2_SO_4_ was added, and the optical density was read at 450 nm using a microplate reader.

### Surface and intracellular staining and FACS analysis

Surface and intracellular staining of CD4 + T cells was performed to evaluate T-bet or RORγt. Cells were stained with anti-CD4-peridinin chlorophyll/cyanin 5.5 (PerCP/Cy5.5) (BioLegend, CA, USA) extracellularly, and fixed and permeabilized with forkhead box protein 3 (Foxp3)/transcription factor, fixation/permeabilization concentrate, and diluent (Thermo Fisher Scientific, MA, USA). Anti-T-bet-allophycocyanin (APC) (BioLegend, CA, USA) and anti-RORγt- phycoerythrin (PE) (Becton Dickinson, NJ, USA) were used for the intracellular staining.

Surface and intracellular staining of CD4 + T cells was performed to evaluate CD25 and Foxp3. Cells were stained with anti-CD4-PerCP/Cy5.5 and anti-CD25-APC (BioLegend, CA, USA) extracellularly, and fixed and permeabilized as mentioned above. Anti-Foxp3-Alexa Fluor (AF488) (BioLegend, CA, USA) was used for the intracellular staining.

Surface staining of CD4 + T cells was performed to evaluate CXCR5 or ICOS. First, the cells were stained with anti-CD4-PerCP/Cy5.5 and anti-ICOS-APC (BioLegend, CA, USA) and incubated with biotin-conjugated anti-CXCR5 (Becton Dickinson, NJ, USA). Next they were stained with PE-conjugated streptavidin.

For intracellular staining of cytokines, cells were cultured for 4 h in the presence of 50 ng/ml of phorbol myristate acetate (PMA), 1 μg/ml of ionomycin, and 1 μg/ml of GolgiStop (Becton Dickinson, NJ, USA). First, dead cells were stained with fixable viability dye eFlour 780. Next the cells were stained with anti-CD4-PerCP/Cy5.5 extracellularly and fixed and permeabilized as described above. Anti-IFNγ-fluorescein isothiocyanate (FITC) (BioLegend, CA, USA), anti-T-bet- APC, anti-IL-17A-phycoerythrin/cyanin 7 (PE/Cy7) (BioLegend, CA, USA), and anti-RORγt-PE was used for the intracellular cytokine staining.

Samples were analyzed with a FACSVerse flow cytometer (Becton Dickinson, NJ, USA), and the data were analyzed with FlowJo software (Tree Star, OR, USA).

### Crisscross coculture with CD4 + T cells and CD11c + dendritic cells

CD4 + T cells and CD11c + dendritic cells (DCs) in the draining LNs were isolated by positive selection using the magnetic activated cell sorting (MACS) system with anti-CD4 monoclonal antibody (mAb) and anti-CD11c mAb (Miltenyi Biotec, Bergisch Gladbach, Germany) respectively. The prepared cells were 98.8 ± 0.15% pure CD4 + T cells and 97.4 ± 0.35% pure CD11c + DCs.

1 × 10^5^ CD4 + T cells and 2 × 10^4^ CD11c + DCs were cultured in the presence of 100 μg/mL of denatured CII for 96 h. Culture medium was RPMI 1640 medium (Sigma-Aldrich, MO, USA) containing 10% fetal bovine serum (FBS), 100 units/mL of penicillin, 100 μg/mL of streptomycin, and 50 μM 2-mercaptoethanol.

### Analysis of CII-reactive cytokine production

The supernatants were collected after 96 h of cell culture with CII. The cytokine levels were analyzed using the LEGENDplex bead-based immunoassay (BioLegend, CA, USA), according to the protocol supplied by the manufacturer. In brief, 20 μl of assay buffer, standard or sample, and mixed capture beads was added to the wells of a 96-well plate and the plate was shaken at 800 rpm for 2 h. The beads were washed with 200 μl of wash buffer. 20 μl of detection antibodies was added to the wells and the plate was shaken at 800 rpm for 1 h. Then, 20 μl of PE-conjugated streptavidin was added to the wells and the plate was shaken at 800 rpm for 30 min. After washing the beads with wash buffer, the standard and samples were read on a FACSVerse flow cytometer, and the data were analyzed with the software provided by BioLegend.

### Quantitative real-time polymerase chain reaction (qRT-PCR)

Total ribonucleic acid (RNA) was extracted with Isogen (Nippon Gene, Tokyo, Japan) according to the instructions provided by the manufacturer. Reverse transcription was used to obtain complementary DNA (cDNA) with a commercially available kit (TaKaRa Bio, Otsu, Japan). A TaqMan Assay-on-Demand gene expression product (Thermo Fisher Scientific, MA, USA) was used for primers and qRT-PCR was performed with QuantStudio 3 (Thermo Fisher Scientific, MA, USA). The expression levels were normalized relative to the expression of *gapdh*.

### Isolation of naïve CD4 + T cells

Single cell suspensions from the spleen were prepared, and CD4 + CD62L + naïve T cells were isolated using a CD4 + CD62L + T cell MACS cell solation kit (Miltenyi Biotec, Bergisch Gladbach, Germany), according to the instructions provided by the manufacturer. The prepared cells were 95.1 ± 3.5% pure naïve CD4 + T cells, as confirmed via FACS analysis.

### In-vitro Th17 polarization of naïve CD4 + T cells

Naïve CD4 + T cells (2 × 10^5^ cells/well on the 96-well plate) were cultured for 72 h with 2 μg/ml plate-bound anti-CD3ε mAb, 1 μg/ml soluble anti-CD28, 2.5 ng/ml human TGF-β, 50 ng/ml of mouse IL-6, and 10 ng/ml of mouse IL-23 in the culture medium described above.

### Separation of infiltrating cells of arthritic joints

Arthritic ankle joints were harvested 35 days post first CII immunization and were incubated with 0.08 Wunsch unit/ml of Liberase at 37 °C for 1 h. Muscles or connective tissues surrounding the ankle joint was removed manually and ankle joint capsule was cut open. The joint capsule was washed with culture medium suspending the infiltrating cells.

### RNA sequencing

CD4 + T cells in the draining LNs 10 days post first CII immunization were isolated using MACS. Total RNA was extracted from ten-thousand cells using TRIzol (Thermo Fisher Scientific, MA, USA). NEBNext rRNA Depletion Kit (New England Biolabs, MA, USA) was used for rRNA-depletion with 500 ng of total RNA. This was followed by directional library synthesis using the NEBNext Ultra Directional RNA Library Prep Kit. The libraries were sequenced with Illumina NextSeq500 (illumina, CA, USA). The reads were mapped to reference with gene and transcript annotation using Ensemble gene annotation system. Total count for each gene was normalized using the quantile method and was used to perform principal component analysis, to generate the scatter plot, and to plot the heatmap with Morpheus (provided by the Broad Institute). Gene Ontology analysis was performed using DAVID (Laboratoy of Human Retrovirology and Informatics).

### Statistical analysis

Statistical analysis was performed using IBM SPSS statistics version 25 (IBM, NY, USA). Data are expressed as mean ± standard error of the mean (SEM). Differences between groups were examined for statistical significance using the Welch’s t-test, Dunnett’s test or Log-rank test. *P* values less than 0.05 were considered significant.

## Supplementary Information

Supplementary Information.

## Data Availability

RNA sequencing data are available at the DNA Data Bank of Japan (DDBJ) under DRA accession number: DRA011788.

## References

[1] Szabo SJ (2000). A novel transcription factor, T-bet, directs Th1 lineage commitment. Cell.

[2] Ivanov II (2006). The orphan nuclear receptor RORγt directs the differentiation program of proinflammatory IL-17+ T helper cells. Cell.

[3] Kawashima M, Miossec P (2005). mRNA quantification of T-bet, GATA-3, IFN-γ, and IL-4 shows a defective Th1 immune response in the peripheral blood from rheumatoid arthritis patients: Link with disease activity. J. Clin. Immunol..

[4] Leipe J (2010). Role of Th17 cells in human autoimmune arthritis. Arthritis Rheum..

[5] Chu CQ, Song Z, Mayton L, Wu B, Wooley PH (2003). IFNγ deficient C57BL/6 (H-2b) mice develop collagen induced arthritis with predominant usage of T cell receptor Vβ6 and Vβ8 in arthritic joints. Ann. Rheum. Dis..

[6] Guedez YB (2001). Genetic ablation of interferon-γ up-regulates interleukin-1β expression and enables the elicitation of collagen-induced arthritis in a nonsusceptible mouse strain. Arthritis Rheum..

[7] Chu CQ, Swart D, Alcorn D, Tocker J, Elkon KB (2007). Interferon-γ regulates susceptibility to collagen-induced arthritis through suppression of interleukin-17. Arthritis Rheum..

[8] Corneth OBJ (2014). Absence of interleukin-17 receptor a signaling prevents autoimmune inflammation of the joint and leads to a Th2-like phenotype in collagen-induced arthritis. Arthritis Rheumatol..

[9] Kondo Y (2012). Overexpression of T-bet gene regulates murine autoimmune arthritis. Arthritis Rheum..

[10] Yokosawa M (2017). T-bet over-expression regulates aryl hydrocarbon receptor-mediated T helper type 17 differentiation through an interferon (IFN)γ independent pathway. Clin. Exp. Immunol..

[11] Plank MW (2017). Th22 cells form a distinct Th lineage from Th17 cells in vitro with unique transcriptional properties and Tbet-dependent Th1 plasticity. J. Immunol..

[12] Ciofani M (2012). A validated regulatory network for Th17 cell specification. Cell.

[13] Zhou L, Littman DR (2009). Transcriptional regulatory networks in Th17 cell differentiation. Curr. Opin. Immunol..

[14] Stadhouders R, Lubberts E, Hendriks RW (2018). A cellular and molecular view of T helper 17 cell plasticity in autoimmunity. J. Autoimmun..

[15] Morgan ME (2003). CD25+ cell depletion hastens the onset of severe disease in collagen-induced arthritis. Arthritis Rheum..

[16] Morgan ME (2005). Effective treatment of collagen-induced arthritis by adoptive transfer of CD25+ regulatory T cells. Arthritis Rheum..

[17] Nakae S, Nambu A, Sudo K, Iwakura Y (2003). Suppression of immune induction of collagen-induced arthritis in IL-17-deficient mice. J. Immunol..

[18] Ryu JG (2015). Treatment of IL-21R-Fc control autoimmune arthritis via suppression of STAT3 signal pathway mediated regulation of the Th17/Treg balance and plasma B cells. Immunol. Lett..

[19] Leavenworth JW, Wang X, Schellack C, Spee P, Cantor H (2011). Mobilization of natural killer cells inhibits development of collagen-induced arthritis. Proc. Natl. Acad. Sci. U. S. A..

[20] Zhang H (2020). An IL-27-driven transcriptional network identifies regulators of IL-10 expression across T helper cell subsets. Cell Rep..

[21] McGeachy MJ (2007). TGF-β and IL-6 drive the production of IL-17 and IL-10 by T cells and restrain TH-17 cell-mediated pathology. Nat. Immunol..

[22] Sun M (2019). RORγt represses IL-10 production in Th17 cells to maintain their pathogenicity in inducing intestinal inflammation. J. Immunol..

[23] Cho YG, Cho ML, Min SY, Kim HY (2007). Type II collagen autoimmunity in a mouse model of human rheumatoid arthritis. Autoimmun. Rev..

[24] Nakayamada S (2011). Early Th1 cell differentiation is marked by a Tfh cell-like transition. Immunity.

[25] Djuretic IM (2007). Transcription factors T-bet and Runx3 cooperate to activate Ifng and silence Il4 in T helper type 1 cells. Nat. Immunol..

[26] Svensson L, Nandakumar KS, Johansson Å, Jansson L, Holmdahl R (2002). IL-4-deficient mice develop less acute but more chronic relapsing collagen-induced arthritis. Eur. J. Immunol..

[27] Mitsdoerffer M (2010). Proinflammatory T helper type 17 cells are effective B-cell helpers. Proc. Natl. Acad. Sci. U. S. A..

[28] Nazeri S, Zakeri S, Mehrizi AA, Sardari S, Djadid ND (2020). Measuring of IgG2c isotype instead of IgG2a in immunized C57BL/6 mice with *Plasmodium vivax* TRAP as a subunit vaccine candidate in order to correct interpretation of Th1 versus Th2 immune response. Exp. Parasitol..

[29] Yang XO (2008). T helper 17 lineage differentiation is programmed by orphan nuclear receptors RORα and RORγ. Immunity.

[30] Zhang F, Meng G, Strober W (2008). Interactions among the transcription factors Runx1, RORγt and Foxp3 regulate the differentiation of interleukin 17-producing T cells. Nat. Immunol..

[31] Lazarevic V (2011). T-bet represses TH 17 differentiation by preventing Runx1-mediated activation of the gene encoding RORγt. Nat. Immunol..

[32] Fossiez F (1996). T cell interleukin-17 induces stromal cells to produce proinflammatory and hematopoietic cytokines. J. Exp. Med..

[33] Chabaud M, Page G, Miossec P (2001). Enhancing effect of IL-1, IL-17, and TNF-α on macrophage inflammatory protein-3α production in rheumatoid arthritis: Regulation by soluble receptors and Th2 cytokines. J. Immunol..

[34] Huber AR, Kunkel SL, Todd RF, Weiss SJ (1991). Regulation of transendothelial neutrophil migration by endogenous interleukin-8. Science.

[35] Hirota K (2007). Preferential recruitment of CCR6-expressing Th17 cells to inflamed joints via CCL20 in rheumatoid arthritis and its animal model. J. Exp. Med..

[36] Sato K (2006). Th17 functions as an osteoclastogenic helper T cell subset that links T cell activation and bone destruction. J. Exp. Med..

[37] Kotake S (1999). IL-17 in synovial fluids from patients with rheumatoid arthritis is a potent stimulator of osteoclastogenesis Find the latest version: IL-17 in synovial fluids from patients with rheumatoid arthritis is a potent stimulator of osteoclastogenesis. J. Clin. invest.

[38] Wang J (2006). Transcription factor T-bet regulates inflammatory arthritis through its function in dendritic cells. J. Clin. Invest..

[39] Joshi NS (2007). Inflammation directs memory precursor and short-lived effector CD8+ T cell fates via the graded expression of T-bet transcription factor. Immunity.

[40] Ehinger M (2001). Influence of CD4 or CD8 deficiency on collagen-induced arthritis. Immunology.

[41] Gao XM, McMichael AJ (1996). Cytotoxic T lymphocytes specific for murine type II collagen do not trigger arthritis in B10 mice. Clin. Exp. Immunol..

[42] Liu N, Ohnishi N, Ni L, Akira S, Bacon KB (2003). CpG directly induces T-bet expression and inhibits IgG1 and IgE switching in B cells. Nat. Immunol..

[43] Coffman RL, Sher A, Seder RA (2010). Vaccine adjuvants: Putting innate immunity to work. Immunity.

[44] Huber K, Sármay G, Kövesdi D (2016). MZ B cells migrate in a T-bet dependent manner and might contribute to the remission of collagen-induced arthritis by the secretion of IL-10. Eur. J. Immunol..

[45] Finotto S (2002). Development of spontaneous airway changes consistent with human asthma in mice lacking T-bet. Science.

[46] Bendele A (1999). Efficacy of sustained blood levels of interleukin-1 receptor antagonist in animal models of arthritis: Comparison of efficacy in animal models with human clinical data. Arthritis Rheum..

