# Supplementary material for: T-bet represses collagen-induced arthritis by suppressing Th17 lineage commitment through inhibition of RORγt expression and function
Source: Sci Rep. 2021 Aug 30;11:17357. doi: 10.1038/s41598-021-96699-5 (PMC8405656; doi:10.1038/s41598-021-96699-5)
Supplement: Supplementary file 1 — Supplementary Information. [file 41598_2021_96699_MOESM1_ESM.pdf]

T-bet represses collagen-induced arthritis by suppressing Th17 lineage commitment through inhibition of RORγt expression and function

Masaru Shimizu[1], Yuya Kondo[1], Reona Tanimura[1], Kotona Furuyama[1], Masahiro Yokosawa[1], Hiromitsu Asashima[2], Hiroto Tsuboi[1], Isao Matsumoto[1], Takayuki Sumida[1]*

1. Department of Internal Medicine, Faculty of Medicine, University of Tsukuba, 1-1-1 Tennodai, Tsukuba City, Ibaraki 305-8575, Japan.

2. Departments of Neurology and Immunobiology, Yale School of Medicine, New Haven, CT 06520, USA.

* Corresponding author.

Takayuki Sumida, MD, PhD

Department of Internal Medicine, Faculty of Medicine, University of Tsukuba

Address: 1-1-1 Tennodai, Tsukuba City, Ibaraki 305-8575, Japan.

Phone and Fax: +81-29-853-3186

E-mail: tsumida@md.tsukuba.ac.jp

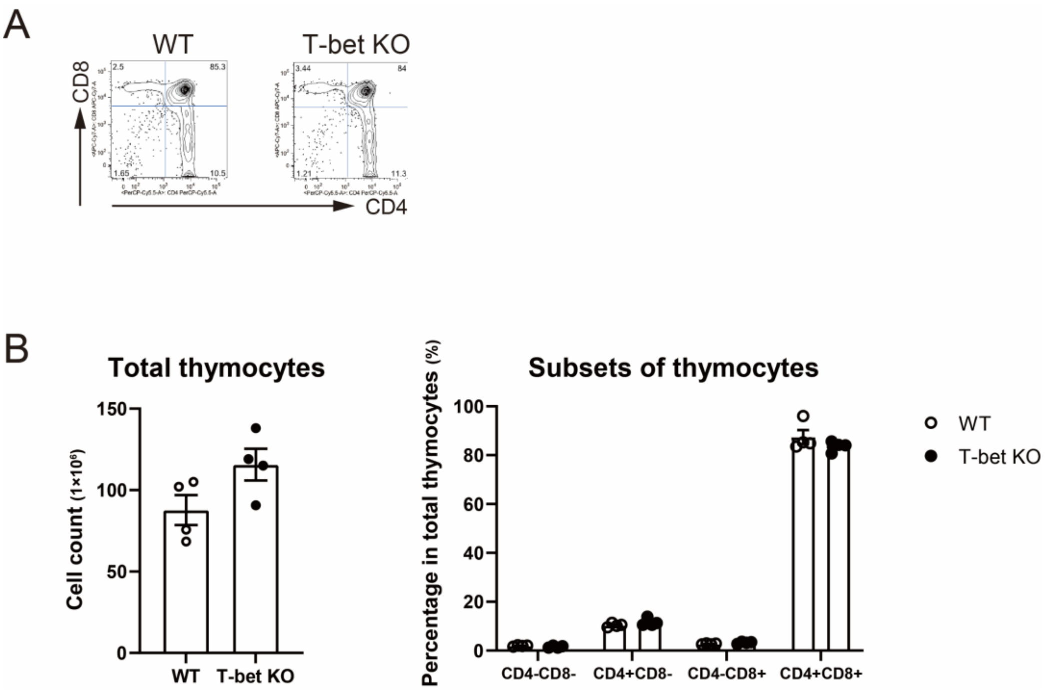

Supplementary Figure 1. Development of T cells in the thymus was normal in T-bet KO mice.

Single cell suspensions of thymus from WT mice (n = 4) and T-bet KO mice (n = 4) were prepared. (A and B) the percentage of each T precursor cells was analyzed using FACS. (A) was generated using FlowJo (version 8.8.7, https://www.flowjo.com/), and (B) was generated using GraphPad Prism 9 (version 9.1.2, https://www.graphpad.com/scientific-software/prism/).

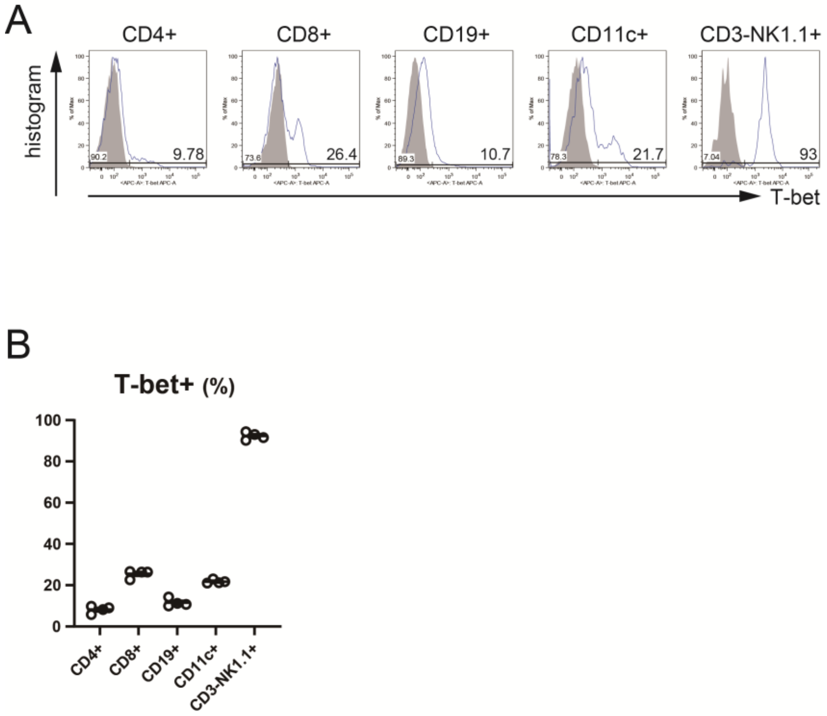

Supplementary Figure 2. T-bet expression in the immune system after CII immunization

WT mice (n = 4) and T-bet KO mice (n = 4) were immunized with CII and their draining LNs were harvested at day 10. (A and B) The percentage of T-bet+ cells in each immune cell subset from the draining LNs of WT mice was analyzed using FACS. (A) was generated using FlowJo (version 8.8.7, https://www.flowjo.com/), and (B) was generated using GraphPad Prism 9 (version 9.1.2, https://www.graphpad.com/scientific-software/prism/).

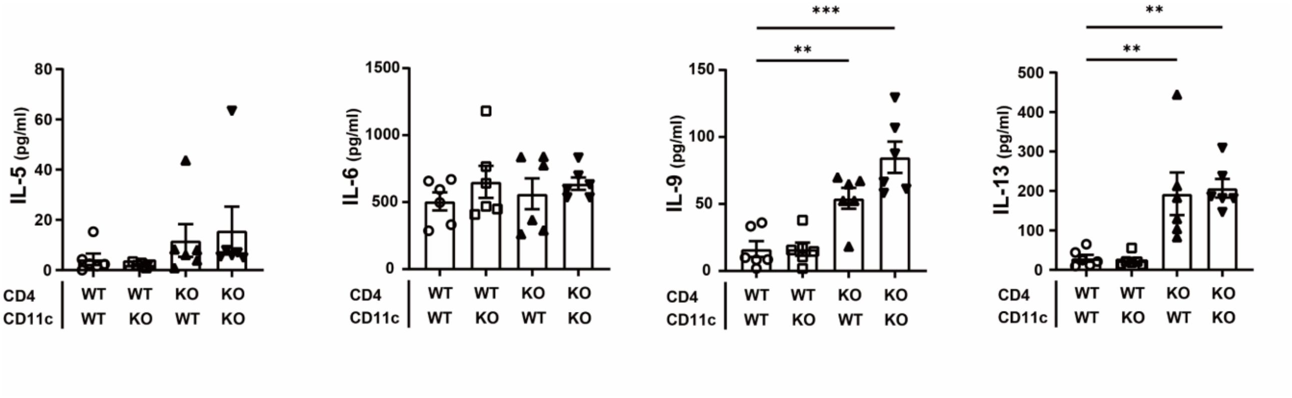

Supplementary Figure 3. CII-reactive cytokine production from CD4+ T cells

At day 10 post first CII immunization, CD4+ T cells and CD11c+ dendritic cells were purified from the draining LNs of WT mice (n = 6) and T-bet KO mice (n = 6) and cultured with 100 μg/mL of denatured CII for 96 hours. Levels of the cytokines in supernatants were measured using multiplex cytokine assay. The origin of CD4+ T cells and CD11c+ dendritic cells is indicated below the bar graphs. Data are presented as mean ± SEM. Statistical tests: one-way ANOVA with Dunnett's post hoc test. (**P* < 0.05, ***P* < 0.01, ****P* < 0.001)

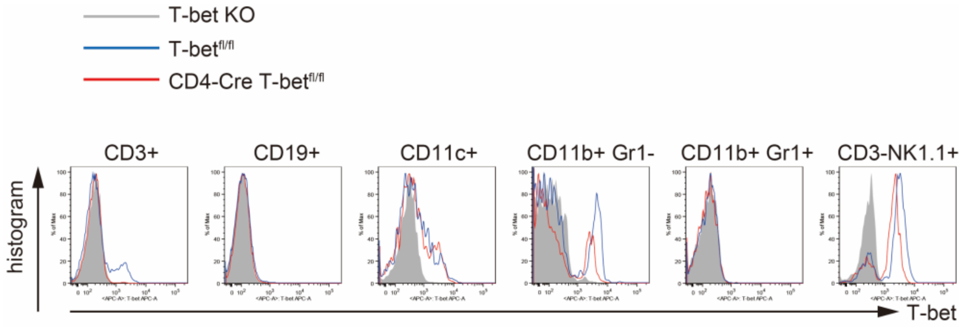

Supplementary Figure 4. Generation of mice with T-cell specific T-bet ablation

C57BL/6 T-bet$^{fl/fl}$ (T-bet$^{fl/fl}$) mice were crossed to C57BL/6 CD4-Cre mice to generate C57BL/6 CD4-Cre T-bet$^{fl/fl}$ (cKO) mice. WT mice, cKO mice, and T-bet KO mice were sacrificed, and their spleens were harvested. The percentage of T-bet+ cells in each subset of splenocytes was analyzed using FACS. The FACS plots were generated using FlowJo (version 8.8.7, https://www.flowjo.com/).

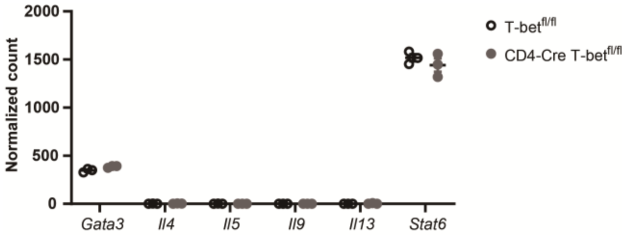

Supplementary Figure 5. The expression levels of Th2 cell-related genes in CD4+ T cells from T-bet$^{fl/fl}$ mice and cKO mice were comparable

At day 10 post first immunization with CII, CD4+ T cells were isolated from the draining LNs of T-bet$^{fl/fl}$ mice (n = 3) and CD4-Cre T-bet$^{fl/fl}$ (cKO) mice (n = 3) and the transcriptome was analyzed. Expression levels of the Th2 cell-related genes were shown. The figure was generated using GraphPad Prism 9 (version 9.1.2, https://www.graphpad.com/scientific-software/prism/). For pairwise analysis, edgeR analysis was performed.

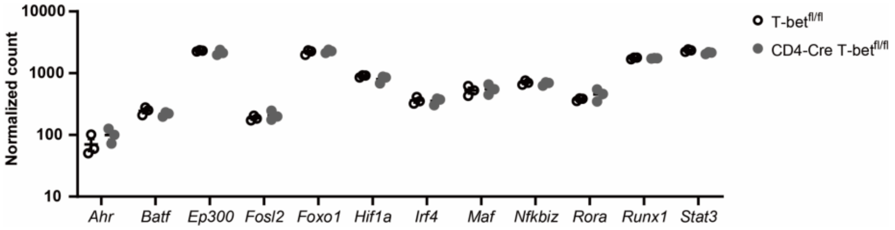

Supplementary Figure 6. T-bet had no effect on the expression levels of the genes responsible for regulating *rorc* or *il17a*

At day 10 post first immunization with CII, CD4+ T cells were isolated from the draining LNs of T-bet$^{fl/fl}$ mice (n = 3) and CD4-Cre T-bet$^{fl/fl}$ (cKO) mice (n = 3) and the transcriptome was analyzed. Expression levels of the genes known to regulate *rorc* or *il17a* were shown. The figure was generated using GraphPad Prism 9 (version 9.1.2, https://www.graphpad.com/scientific-software/prism/). For pairwise analysis, edgeR analysis was performed.
